# Alternatives to the face-to-face consultation in general practice: focused ethnographic case study

**DOI:** 10.3399/bjgp18X694853

**Published:** 2018-01-30

**Authors:** Helen Atherton, Heather Brant, Sue Ziebland, Annemieke Bikker, John Campbell, Andy Gibson, Brian McKinstry, Tania Porqueddu, Chris Salisbury

**Affiliations:** Warwick Medical School, University of Warwick, Coventry.; Centre for Academic Primary Care, University of Bristol, Bristol.; Nuffield Department of Primary Care Health Sciences, University of Oxford, Oxford.; Usher Institute of Population Health Sciences and Informatics, University of Edinburgh, Edinburgh.; University of Exeter Collaboration for Academic Primary Care (APEx), University of Exeter, Exeter.; Health and Social Sciences, University of the West of England, Bristol.; Usher Institute of Population Health Sciences and Informatics, University of Edinburgh, Edinburgh.; Nuffield Department of Primary Care Health Sciences, University of Oxford, Oxford.; Centre for Academic Primary Care, University of Bristol, Bristol.

**Keywords:** communication, electronic mail, ethnography, general practice, qualitative research, remote consultation, workload

## Abstract

**Background:**

NHS policy encourages general practices to introduce alternatives to the face-to-face consultation, such as telephone, email, e-consultation systems, or internet video. Most have been slow to adopt these, citing concerns about workload. This project builds on previous research by focusing on the experiences of patients and practitioners who have used one or more of these alternatives.

**Aim:**

To understand how, under what conditions, for which patients, and in what ways, alternatives to face-to-face consultations present benefits and challenges to patients and practitioners in general practice.

**Design and setting:**

Focused ethnographic case studies took place in eight UK general practices between June 2015 and March 2016.

**Method:**

Non-participant observation, informal conversations with staff, and semi-structured interviews with staff and patients were conducted. Practice documents and protocols were reviewed. Data were analysed through charting and the ‘one sheet of paper’ mind-map method to identify the line of argument in each thematic report.

**Results:**

Case study practices had different rationales for offering alternatives to the face-to-face consultation. Beliefs varied about which patients and health issues were suitable. Co-workers were often unaware of each other’s practice; for example, practice policies for use of e-consultations systems with patients were not known about or followed. Patients reported benefits including convenience and access. Staff and some patients regarded the face-to-face consultation as the ideal.

**Conclusion:**

Experience of implementing alternatives to the face-to-face consultation suggests that changes in patient access and staff workload may be both modest and gradual. Practices planning to implement them should consider carefully their reasons for doing so and involve the whole practice team.

## INTRODUCTION

There is international interest in the potential role of different forms of communication technology to provide an alternative to the face-to-face consultation in health care, with several countries such as Denmark and the US routinely offering these in primary care settings.^1^^–^3 In the UK, policymakers have suggested that alternatives such as telephone, email, or internet video used in the general practice setting could have a transformative impact, alleviating staff workload and improving patient access.^4^^,^^5^

Despite the push to introduce alternatives to the face-to-face consultation, most general practices have been slow to adopt their use,^6^ citing concerns about their potential impact, particularly on workload and the need to ensure patient safety.^7^^,^^8^ However, some types of consultation are more embedded than others: telephone consultations have been used in general practice for some time, with more known about their use than for email or internet video.

Studies have attempted to assess the impact of alternatives to the face-to-face consultation on consultation numbers and patient satisfaction in primary care settings^9^^–^12 but these studies are of limited number and in some cases are of low quality.^13^ Studies about the use of email consultation have assessed impact in the context of a patient portal that offers several functions, for example, appointment booking and access to medical records,^14^^,^^15^ making it difficult to draw conclusions about the impact of the alternative to the face-to-face consultation alone. Recent years have seen a plethora of small and local pilot projects and commercial initiatives around specific systems,^16^^,^^17^ which proliferate in an environment of patchy and inconclusive evidence.

Patients, practice staff, and policymakers may have different aims in encouraging alternatives to face-to-face consultations. However, it is not always clear which hypotheses underpin the mechanisms through which alternative methods of consultation might lead to benefits.^8^^,^^18^^–^20 The ‘Alt-Con’ project (alternatives to face-to-face consultation in general practice) explores these issues and builds on previous research^7^^,^^21^^,^^22^ by focusing on the experiences, rather than the hypothetical perspectives, of patients and practitioners who have used an alternative to the face-to-face consultation. The aim of the study was to understand how, under what conditions, for which patients, and in what ways, alternatives to face-to-face consultations present benefits and challenges to patients and practitioners in general practice. In particular, the study aimed to explore the feasibility and impact of alternatives to face-to-face consultations, from the perspectives of both staff and patients.

**How this fits in**

Enthusiasts have led the introduction of alternatives to the face-to-face consultation in general practice though uptake has been patchy and practices have concerns about being inundated by patients. Patients like them and find them convenient on the whole. By conducting observations as well as interviews with all staff groups and patients who have used an alternative to the face-to-face consultation, this study has obtained insights into the varied rationale for their introduction and expanded the evidence on how they work in practices with recent experience of trying to implement them. There is an expectation that practices will ‘go digital’ to help manage demand and this study suggests that any decision should be a considered one, in particular thinking about the rationale for introduction, what the practice hopes to gain, and whether there is evidence that alternative consultation forms will achieve these aims.

## METHOD

The Alt-Con Project included an initial scoping survey of general practices^6^ and a conceptual review^23^ to inform a mixed methods evaluation using a case study design and combining quantitative and qualitative methods. The qualitative element of the study is reported here; focused ethnographic case studies were conducted in eight general practices in the UK between June 2015 and March 2016. In a team-based focused ethnography, rather than embedding a researcher in a social setting for a lengthy period, more targeted data collection is used to explore the study topics.^24^ The rationale for using this approach is described in detail elsewhere.^25^ The case studies were focused on use of alternatives to face-to-face consultations in each practice, which included telephone consultations, email, ‘e-consultations’ (www.econsult.net/ and www.askmygp.uk/), and internet video (for example, Skype™). Telephone consultations used specifically for triage were excluded as a parallel study explores this model of care.^26^ The final report for the funder on the wider study will be published in 2018.^27^ This study reports findings from the ethnography, in line with standards for reporting qualitative research.^28^

### Theoretical approach

Weiss’s theory-based evaluation approach was used.^29^ Weiss distinguishes between ‘program theory’, which specifies the mechanism of change, and ‘implementation theory’, which describes how the intervention is carried out. To develop the ‘program theory’ a realist approach^30^ was used to understand provision of alternatives to face-to-face consultations in terms of context, how and why alternatives to the face-to-face consultation might lead to benefits and challenges (mechanism), and what matters to patients and practitioners (outcomes). To contribute to an ‘implementation theory’ the study explored why the case study practices had decided to offer alternatives to face-to-face consultations to different groups of patients and the experiences of practice staff and the organisation.

### Recruitment

A scoping survey^6^ was conducted to identify general practices that were currently providing alternatives to face-to-face consultations in the four areas the study sought to recruit practices: Bristol, Oxfordshire, Lothian, and Highlands & Islands. Practices who identified as having experience of implementing one or more form of alternatives to face-to-face consultations were approached and invited them to participate. Two practices in Scotland and six in England were recruited.

### Data collection

The fieldwork team consisted of five researchers: a day-to-day lead, a senior lead, and three ethnographers working in the field. Each of the field ethnographers were allocated two or three practices and collected data in each practice for up to 8 weeks.

Data were gathered through non-participant observation across the practice in reception areas, communal staff areas, and in consultation rooms, through informal conversations and semi-structured interviews with administration staff and GPs, and interviews with patients. Practice documents and protocols on alternatives to the face-to-face consultations were reviewed. Staff participants for interviews were selected by the ethnographers in the field. Staff assisted in identifying patients to invite to be interviewed. Patients were selected using purposive sampling, ensuring that patients had different characteristics in relation to age, sex, ethnicity, disability, frequency of attendance, and whether or not they had long-term health conditions. Specifically included were people in ‘hard-to-reach’ groups with regard to accessing general practice, for example, young males, the vulnerably housed, and minority ethnic groups. All patients invited to participate in interviews had experience of using an alternative to the face-to-face consultation within the practice.

In preparation for the ethnography, a conceptual review^23^ was conducted to identify areas of focus for enquiry. These included the staff members they should consider observing (for example, focusing on reception staff as well as clinical staff), things they might want to look out for (for example, dynamics within the clinic between staff members), and where they might look (for example, areas where team members interact beyond the consultation areas).

The findings were used to devise a case study guide to help ensure consistency of approach between the three field-workers, who held regular phone conferences and meetings throughout the study. Data were collected using anonymised field notes, which were then transferred into a ‘practice summary’ template to facilitate comparisons between the practices.

Interviews were digitally recorded, using an encrypted recorder. The files were transcribed verbatim by a professional transcription service.

### Case study sample

The eight participating practices had a range of list sizes, from 1938 to 18 353. One was in a rural area, two in semi-rural areas, and five in the inner city. Practices included some from the most and least deprived areas in terms of deprivation deciles. The participating practices used a varied range of alternatives to the face-to-face consultation (Table 1).

**Table 1.** Description of case study sites

**Practice reference**	**Size of practice**	**Location of practice**	**Deprivation categorisation and decile^a^**	**Alternative to the face-to-face consultation used**	**Days spent in observation, *n***
A	18 353 registered patients	Inner city	Deprived 3	Telephone consultations, e-consultations,^b^ isolated use of email	25
B	8954 registered patients	Inner city	Deprived 3	Telephone consultations, isolated use of email	19
C	15 000 registered patients	Inner city	Mixed 4	Telephone consultations, e-consultations,^b^ isolated use of email	18
D	1938 registered patients	Rural	Mixed 5	Telephone, video	8
E	7196 registered patients	Inner city	Deprived 1	Telephone, e-consultations,^b^ isolated use of email	17
F	13 778 registered patients	Semi-rural	Affluent 10	Telephone, email	25
G	13 511 registered patients	Semi-rural	Mixed 6	Telephone, email	16
H	6597 registered patients	Inner city	Affluent 10	Telephone, email	11

a Practices A–C and F–H based on the Index of Multiple Deprivation rank. Practices E and F measured by percentage of practice patients living in data zones defined as the 15% most deprived in Scotland (population weighted).

b All those using e-consults in the study were piloting the use of the software for free.

In total, 45 staff members were interviewed. Staff participants included 19 GPs, 10 practice managers or deputies, one practice coordinator, one nurse practitioner, five practice nurses, one rural health worker, five receptionists, one patient service manager, one practice administrator, and an IT manager. In total, 39 patient and carer participants were also recruited. See Box 1 for participant characteristics.

Box 1. **Patient participants (*N* = 39)**

**Table:** 

13 male, 25 female, 1 transgender. 10 identified as carers. Seven had restricted mobility. 30 had a long-term condition. Six had a mental health condition (where condition was disclosed).^a^ 15 had multimorbidity (where conditions were disclosed).^a^ 16 were educated to degree level or above.

a It was not compulsory to disclose any long-term condition or disability.

### Data analysis

The coding frame was devised by the focused ethnographic team at a face-to-face meeting early in the data collection period. The three ethnographers read and coded interview transcripts and field notes, condensing the field notes into a summary profile. Every transcript and summary profile was read, and the coding was checked to ensure reliability and comparability. Transcripts and summary profiles were then entered into NVivo software (version 10) and a series of NVivo reports were generated, with data from the staff and patient interviews and the field notes integrated. Three team members read all of the reports, applying the ‘one-sheet-of-paper’ mind-mapping method^31^ to identify the line of argument in each thematic report, and between the three researchers a condensed summary (‘one sheet of paper’) was produced for each thematic code. Outliers and negative cases were included in these reports. At this point, the wider study team (including three experienced GPs) were paired with members of the ethnographic team to discuss interpretation of the data. The core messages were presented at a wider team meeting and the analysis refined through discussion among all the team members.

## RESULTS

Practices described the different rationales for introducing an alternative to the face-to-face consultation, the consequences for the organisation and ways of working, staff and patient experiences, ideas about for whom different types of alternatives to the face-to-face consultation are suitable, the barriers and facilitators to implementation, and the outcomes that mattered to the participants.

### Rationale

Rationale given for introducing an alternative to the face-to-face consultation included:

- the desire to be a modern practice and respond to the expectations of busy, time-poor patients;
- the only way of providing health care for patients in remote locations, or with other barriers to attending the practice;
- the acknowledgement that the previous system was broken and unethical in providing a first-come, first-served system that left patients without appointments that they needed;
- the recognition that reception staff and phone lines were overwhelmed; and
- to manage demand and improve efficiency.

Rationales differed between practices, but also within practices; different team members had different perceptions and understanding about the rationale for introducing an alternative to the face-to-face consultation and thus the potential benefit it could bring. In many practices the decision to implement alternative forms of consultation was in the context of a perception of increasing demand and external encouragement from policy to introduce these alternatives. In some practices the decision to implement was triggered by the offer of financial support from the GP Access Fund^32^ for specific project support and free pilots:

*‘Our pilot of* [e-consultation system] *is about to come to an end. We were given this on free trial basis to see what we, and our patients would make of it.’*

(GP1, Practice E, inner city, deprived)

### Practice organisation

In practices without a formal e-consultation system, members of the practice team did not always know whether other staff were, for example, in e-mail contact with their patients. One GP, during an interview, said:

‘I do the same as everyone else in the practice.’

(GP1, Practice C, inner city, mixed)

But accounts from other staff members, and observations by the ethnographer, suggested otherwise.

In the case study practices, policies about emailing patients were either not in place, not known about, or not followed. Contradictions were evident, for example, one GP explained that their practice was trying to discourage patients from engaging in two-way email communication with the practice yet he used email with ‘selected’ (trusted) patients:

*‘What we’re envisaging is … saying, “No reply @ X Medical Practice,” to make it a bit more obvious that you’re not meant to reply*.’

(GP3, Practice F, semi-rural, affluent)

Informal discussions and interviews with staff and patients identified different views about the boundaries of a consultation. Patients described using telephone and email for background information, a perspective that was reinforced if the patient was then asked to attend a face-to-face consultation. Staff were inconsistent in recording consultations in the medical record, for example, it was observed that not all email consultations were necessarily included in the medical record.

### Staff and patient experiences

Patients could only express a preference for an alternative to a face-to-face consultation if they were aware that the practice offered it, and this was not always the case. Telephone consultations were well integrated within the practices studied but the ethnographic observations suggested that patients rarely asked for a non-face-to-face consultation, and receptionists only offered them as a last resort when all appointments were taken. This was consistent with the staff belief *‘that patients prefer to see the doctor’* or, as one of the patients put it, a phone consultation was:

‘… better than nothing, but not 100 per cent.’

(50-year-old female patient, Practice D, rural, mixed)

Other interviews suggested that, depending on the health issues, some patients preferred to avoid coming to the practice.

Depending on how practices organised the working day, alternatives could offer flexibility to both staff and patients. GPs and nurses were able to choose when and in what order to reply to messages or make phone calls:

‘I’m able to manage my time a bit better.’

(GP 2, Practice H, inner city, affluent)

However, the benefit of flexibility was contrasted with the potential for ‘hidden work’ that stretched the working day. One of the practices had examined whether telephone consultations were briefer and had been surprised to find that this was not the case:

‘But we’d thought that we might be able to do two things, two, do two telephone consultations in the time it took to do a face-to-face … and that hasn’t proved to be the case.’

(Practice manager, Practice F, semi-rural, affluent)

It was observed that on some occasions a decision was made to convert to a face-to-face consultation following use of an alternative such as a telephone consultation, and this increased the overall number of consultations with that patient.

### For which patients and problems?

In interviews staff and patients concurred that alternatives to face-to-face consultation might be unsuitable if a new health problem was being presented, if the patient was older or confused, or if the patient was using a complex array of medicines.

Clinicians varied in their views about which patients were most likely to be suitable for an alternative consultation; in some cases these decisions were based on age, socioeconomic status, or ethnic group. One GP commented that telephone consultations were best used with patients who had been born in the UK:

‘I do notice that generally the patients that are born and raised in the UK, you can process their problems quicker.’

(GP1, Practice B, inner city, deprived)

Clinicians felt more confident to gather information via telephone or email if the patient was known to be sensible and deemed to use the system in a judicious manner. Continuity mattered to patients too: for certain health problems, it might be important to know the clinician who would be consulted remotely.

### Implementation

Several barriers and facilitators to implementation were observed. Barriers included difficulties in making patients aware of the option to use an alternative to the face-to-face consultation and subsequently getting them to engage with these alternatives when the face-to-face consultation was still seen as the ‘gold standard’. The lack of understanding within practices about the role of alternatives to the face-to-face consultation and how they might impact on the practice and the staff were also barriers to implementation, with unintended consequences such as an increased workload via conversions to the face-to-face consultation, and potential inequitable delivery of care where clinicians were choosing which patients they would consult with this way.

Receptionists and administrators had a key role in ensuring that new consultation methods were taken up by and delivered to patients, but this was not always acknowledged or considered by other members of the practice. Receptionists were not offered training, and practices were reluctant to invest financially in training for any staff members, sometimes delivering *ad-hoc* or in-house training, or in the case of e-consultation training only the GPs. Training was:

‘… the poor partner, the poor relation.’

(Staff member, Practice F, semi-rural, affluent)

Sometimes factors relating to implementation were addressed up front, for example, the use of ‘out of office’ messages to avoid patients having a long wait for a reply to an email. However, others were not adequately considered beforehand. In one site where video consultation was used there was a lack of facilities, slow computers, and insufficient bandwidth. Video consultations were time consuming to set up and the images were poor. Consequently, consultations defaulted to the telephone. Other structural factors such as not having enough telephone lines, or difficulty in recording via the appropriate systems when a consultation occurred, were also barriers to use. More subtle factors included the impact on professional identity, with the core tenet of general practice being the doctor–patient relationship as conducted in the face-to-face consultation:

‘Medicine’s about relationships really and getting to know your patient as a person.’

(GP3, Practice C, inner city, mixed)

The GP Access Fund^32^ was an important facilitator to implementation, because it provided a rationale, financial support, and training. In several practices the introduction was driven by one or two ‘innovators’ who got alternatives to the face-to-face consultation off the ground. Other facilitators included the identification of a clear role for alternatives to the face-to-face consultation in some conditions and for certain patients. Patients were positive about the use of alternatives to the face-to-face consultation, and both staff and patients shared an understanding about the limitations of these mediums, which made implementation smoother. The flexibility of alternatives to the face-to-face consultation made them easier to ‘slot’ into day-to-day practice. A willingness to adapt their use once introduced was a key facilitator:

‘We created more telephone slots because there was a demand for it.’

(Practice administrator, Practice D, rural, mixed)

### Outcomes that matter to participants

Some clinicians said that they used email to share and gather information when coordinating complex healthcare packages. Nurses explained that they used telephone and email consultations for management of diabetes, for example, for discharge checks and medication reviews. For GPs, the main motivation for introducing alternatives to face-to-face consultation was to help them manage their workload.

Patients said that they liked the efficiency and convenience offered by alternatives to the face-to-face consultation. Some thought that an email that went directly to the GP avoided involving the receptionist in the decision about whether the patient needed to be seen:

*‘Then the decision whether I need to be seen is his* [the GP’s] *… if you phoned the receptionist you haven’t got a hope in hell.”*

(76-year-old male patient with comorbidities, Practice F, semi-rural, affluent)

Beyond these factors, the benefits for patients of alternatives to the face-to-face consultation related to certain elements of the medium, for example, email and e-consultation offered an asynchronous and text-based approach, which was recognised as useful for people who were very anxious, or found face-to-face contact difficult, who had hearing or communication difficulties, and those who struggled to express themselves.

## DISCUSSION

### Summary

Practices introduced alternatives to face-to-face consultation for different reasons and often without a clear rationale for how different forms of consultation might help. Implementation was often not well ‘thought through’ in relation to personnel, training, or logistical factors and which patients were the intended beneficiaries. The intended outcomes were varied, but practice staff emphasised managing patient access while patients emphasised improved convenience and efficiency. There were clear barriers to the implementation of alternatives including structural factors such as insufficient phone lines and poor internet connectivity, and more subtle concerns such as impact on the doctor–patient relationship. Overall uptake of alternatives other than telephone consultations was very low, which may have related to the lack of clear rationale or benefit, inconsistent policies in practices, and structural barriers to use.

### Strengths and limitations

A range of urban and rural practices were included, covering a broad geographical area, and having a wide range of deprivation scores. Each practice used a different combination of alternatives to the face-to-face consultation. Patients from a wide range of ages, health conditions, and socioeconomic groups were interviewed,. One weakness is that just one of the case study practices served a community with a high proportion of patients from ethnic minority backgrounds. Practices using telephone triage systems^26^^,^^27^ where the doctor triages all incoming calls from patients (‘doctor first’) were excluded and it is possible that this may have provided more context for how telephone consultation was used.

Using focused ethnography made it possible to see what people do as well as what they say they do. Team-based focused ethnography is relatively quick (compared with conventional ethnography) and data can be collected at different sites concurrently. This speed is advantageous in research areas where the policy context is constantly changing. However, it does not provide the same depth of exploration as a conventional ethnographic study.

### Comparison with existing literature

Policy documents and reports outline a rationale for the introduction of alternatives to the face-to-face consultation,^5^^,^^33^^,^^34^ but the aims of improving patient access while also controlling practice workload may be in tension. The study has demonstrated that reasons for introduction in practice are varied and sometimes opportunistic rather than carefully considered. Concerns about whether improving access through use of alternatives to the face-to-face consultation will increase rather than decrease workload pressure is evident in much previous research on the use of alternatives such as telephone^35^ or email consultations.^7^ The findings suggest that the impact on patterns and volume of workload is complex and reductions in workload cannot be assumed.

As in this study, earlier studies have described GPs selectively choosing which patients to engage with in email consultations.^7^ The suitability of email for straightforward and uncomplicated questions or conditions was reported in a Danish study.^21^ A conversation analysis of telephone consultations found that patients were less likely to raise additional problems with the GP during a telephone consultation compared with a face-to-face consultation.^36^ This fitted with this study’s finding that patients and doctors were in accord that the telephone was good for ‘basic’ problems or follow-up, but that a face-to-face consultation was needed for more complex problems.

### Implications for research and practice

Technology always has consequences for professional relationships, expectations, and patterns of work. Intervention studies should focus on how these aspects of the service are designed. The feasibility of examining these factors depends on the level of development and current implementation of the technology under investigation.

The low rates of usage of alternative forms of consultation, other than telephone, need further investigation to understand to what extent this relates to a lack of patient awareness or demand, problems of implementation, or simply slow adaptation leading to increased uptake in time.

In order to evaluate alternatives to face-to-face consultations it is necessary to be clearer about the aims and intended outcomes of the initiative. The study has applied the findings reported here and from the wider study^27^ in two ways. First, guidance has been developed and a website resource created for general practices considering the introduction of an alternative to the face-to-face consultation.^37^ Based on this study’s research it outlines five key areas to consider: why do you want to do this, which type of alternative are you interested in, who is it for and why, how do we get it right, and how will we know if it has worked?

Second, the findings have been used to develop a framework and recommendations for future evaluation. Treating provision of alternatives to face-to-face consultations as an intervention, recommendations have been made about the target population, appropriate outcome measures, and best methodological approach for evaluation. This framework will be reported elsewhere.^27^

The findings show that there is a lack of clarity about the match between the different rationales for introducing alternatives to the face-to-face consultation and the intended benefits. Clinicians had varying views on what conditions were suitable for this type of consultation, though agreed that they were not suitable for new or complex conditions. Implementation brings considerable challenges, including the potential for changes in the volume and pattern of workload, implications for the roles of practice staff, and for the equitable delivery of care. When introducing an alternative to the face-to-face consultation the potential for unintended consequences should be considered as these may have a bearing on the potential success of these forms of consultation. However, patients and staff could see potential for benefit from use of a range of types of consultation if these difficulties can be overcome.
